# Supplementary material for: LRRC25 Inhibits IFN-γ Secretion by Microglia to Negatively Regulate Anti-Tuberculosis Immunity in Mice
Source: Microorganisms. 2023 Oct 5;11(10):2500. doi: 10.3390/microorganisms11102500 (PMC10608824; doi:10.3390/microorganisms11102500)
Supplement: Supplementary file 1 [file microorganisms-11-02500-s001.zip › microorganisms-2622454-supplementary.pdf]

Supplementary material

Table S1. The siRNA sequences used in this study.

siRNA	Sequences (5’⟶ 3’)
L1-Sense	GGGCUUUCUUCGACAAGCUTT
L1-Antisense	AGCUUGUCGAAGAAAGCCCTT
L2-Sense	CAGGUUCACAAUGGAUUAUTT
L2-Antisense	AUAAUCCAUUGUGAACCUGTT
L3-Sense	GCCUCAAUUUCAGUGGCCUTT
L3-Antisense	AGGCCACUGAAAUUGAGGCTT
Negative control-Sense	UUCUCCGAACGUGUCACGUTT
Negative control-Antisense	ACGUGACACGUUCGGAGAATT
GAPDH-Sense	CACUCAAGAUUGUCAGCAATT
GAPDH-Antisense	UUGCUGACAAUCUUGAGUGAG

Table S2. The primer sequences used in this study.

Primer	Sequences (5’⟶ 3’)
LRRC25-Forward Primer	GAACTAGGTTGCTGTGGTTATGT
LRRC25-Reverse Primer	GTCTGAGTCCAGTCTACCCTG
ISG15-Forward Primer	GGTGTCCGTGACTAACTCCAT
ISG15-Reverse Primer	CTGTACCACTAGCATCACTGTG
β-actin-Forward Primer	GTGACGTTGACATCCGTAAAGA
β-actin-Reverse Primer	GCCGGACTCATCGTACTCC
IL-1β-Forward Primer	GAAATGCCACCTTTTGACAGTG
IL-1β-Reverse Primer	TGGATGCTCTCATCAGGACAG
IL-12-Forward Primer	CAATCACGCTACCTCCTCTTTT
IL-12-Reverse Primer	CAGCAGTGCAGGAATAATGTTTC
P65-Forward Primer	TGCGATTCCGCTATAAATGCG
P65-Reverse Primer	ACAAGTTCATGTGGATGAGGC

Table S3. Q-PCR of LRRC25 in BV2, C8-D1A and HT22 cells *infected by Mtb for 4 h at an MOI of 5.*

Cell	Ct	$2^{-\Delta\Delta Ct}$	P value	Up or Down
	24.540	1.907653056		
BV2	24.747	1.652678052	P<0.01	Up
	24.536	1.913374077		
	35.486	—		
C8-D1A	36.351	—	P>0.05	None
	36.989	—		
	34.181	0.237303568		
HT22	32.968	0.550074959	P<0.01	Down
	33.789	0.414560168		

Up: Upregulated expression; None: Unexpressed; Down: Downregulated expression.

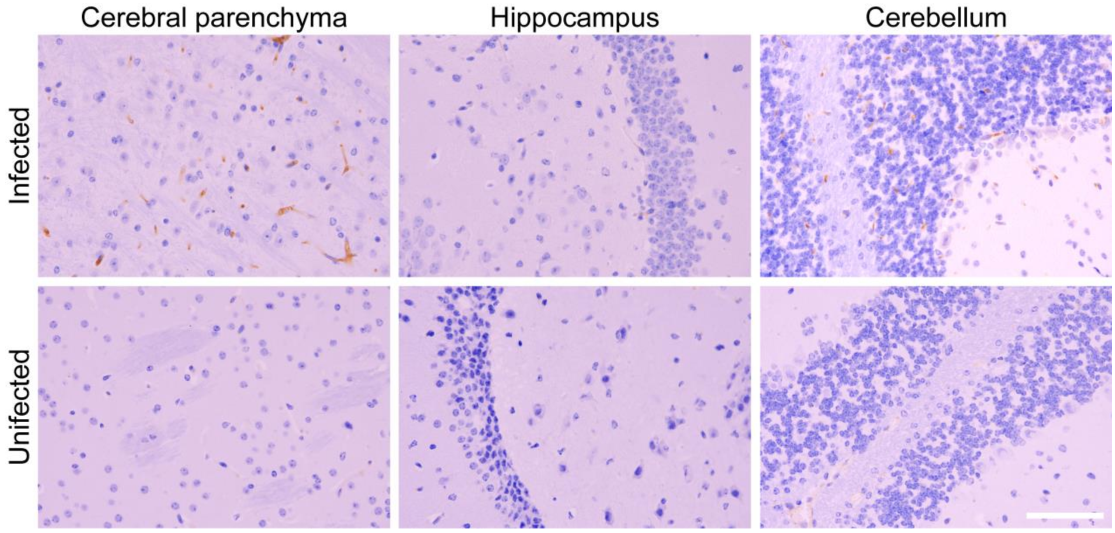

Figure S4. Immunohistochemical images of LRRC25 in cerebrum and cerebellum of mice with tuberculous meningitis. The infected group is mice infected with H37Rv for four weeks. Scale bar, 50 μm.

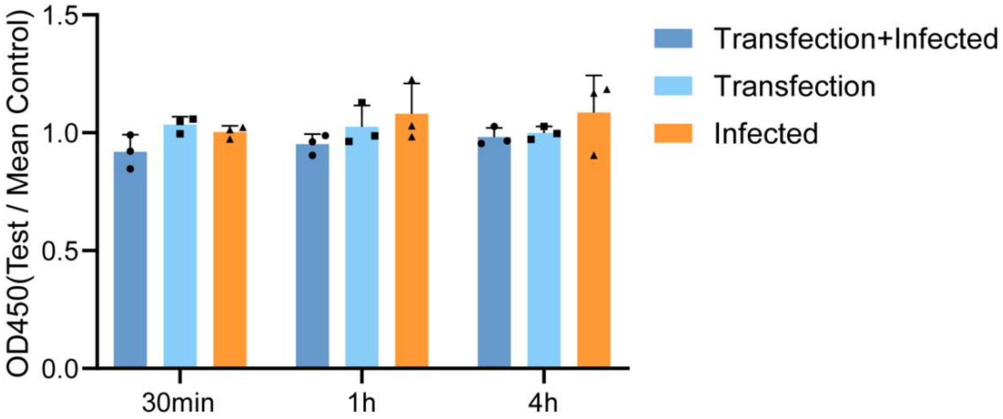

Figure S5. Comparison of cell viability in every group. Cell viability of each group. Transfection: Cells were transfected with L1 for 72 h, Infection: H37Rv infected cells for 4 h, and the MOI was 5. The absorbance (OD450) was measured at 30 min, 1 h and 4 h after adding CCK-8, and the results were expressed as the ratio of the absorbance test group to the mean absorbance negative control group. Bar graphs show the mean ± SD form three independent experiments.

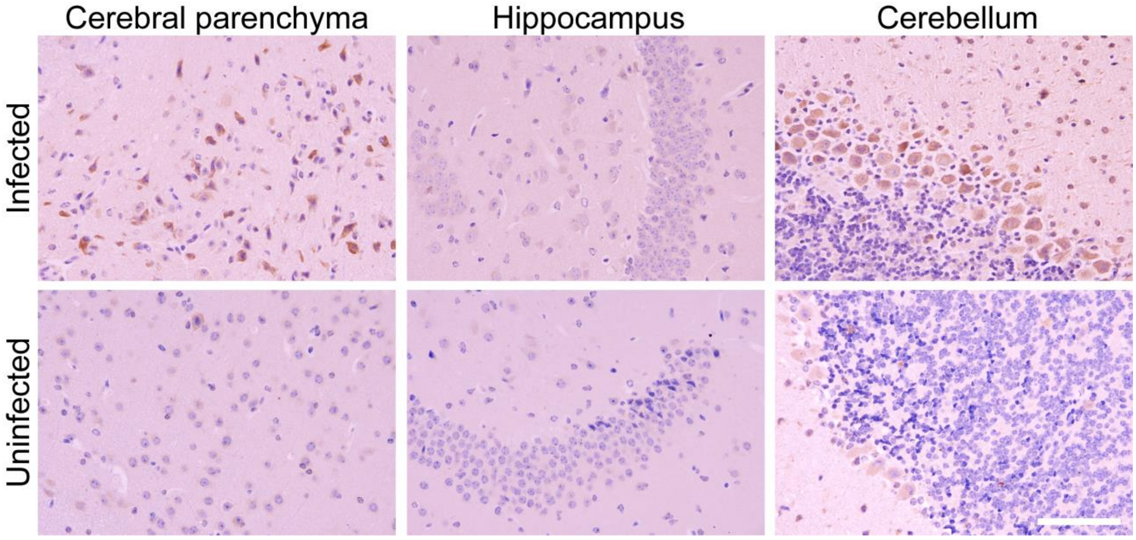

Figure S6. Immunohistochemical images of IL-1β in cerebrum and cerebellum of mice with tuberculous meningitis. The infected group is mice infected with H37Rv for four weeks. Scale bar, 50 μm.

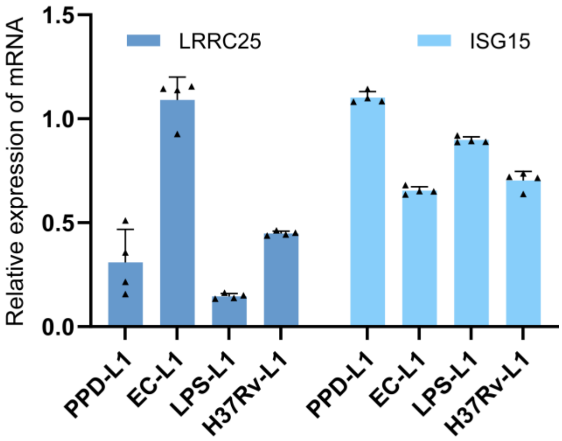

Figure S7. mRNA expression of LRRC25 and ISG15 in microglia stimulated by different *Mtb*-related stimuli. BV2 cells were stimulated with 25 µg/µL PPD (pure protein derivative of *Mtb*), 25 µg/µL EC (EAST-6 and CFP10: early secretory antigen target 6 of *Mtb* and protein 10 of *Mtb* culture filtrate) and 1.5 µg/µL LPS (lipopolysaccharide), and BV2 cells were treated with L1 for 72 h. Bar graphs show the mean ± SD form four independent experiments.

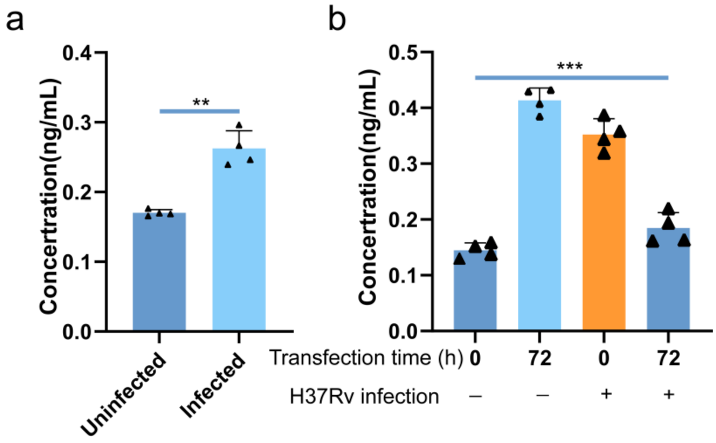

Figure S8. Secretion of IL-12 in the cell supernatant of different groups. (a) IL-12 expression in BV2 cells in the cell culture supernatant before and after tuberculosis infection, Wilcoxon-Ruskal-Wallis test. (b) IL-12 levels in the cell supernatants of the H37Rv-infected and uninfected groups after transfection for 48 h and 72 h, Two-way ANOVA. Bar graphs show the mean ± SD form four independent experiments. *$P < 0.05$, **$P < 0.01$, ***$P < 0.001$.

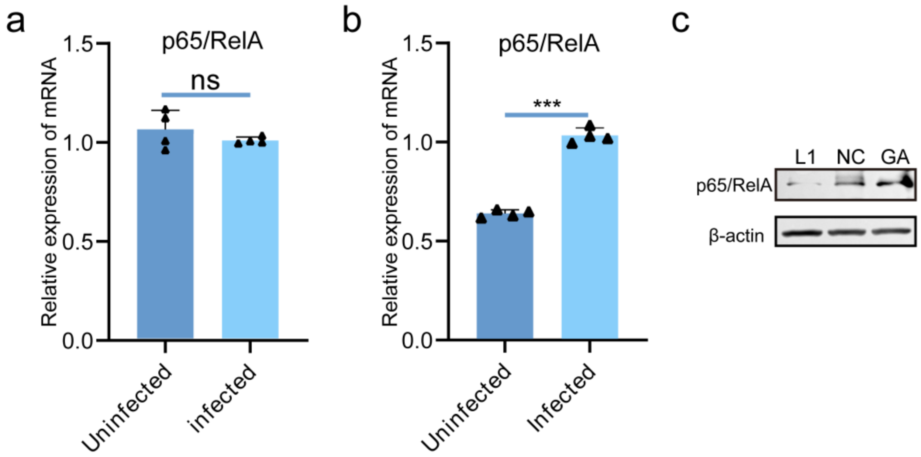

Figure S9. LRRC25 interacts with p65/RelA in NF-κB pathway. (a) The mRNA relative expression of p65/RelA in BV2 cells infected by *Mtb*, Wilcoxon-ruskal-Wallis test. (b) After LRRC25 was silenced for 72 h, the mRNA relative expression of p65/RelA in BV2 cells infected by *Mtb* was observed, Wilcoxon-Ruskal-Wallis test. (c) After LRRC25 was silenced for 72 h, the protein expression of

p65/RelA in BV2 cells infected with *Mtb*. L1: LRRC25 silenced group. Bar graphs show the mean ± SD form four independent experiments, Student's t test. *$P < 0.05$, **$P < 0.01$, ***$P < 0.001$.

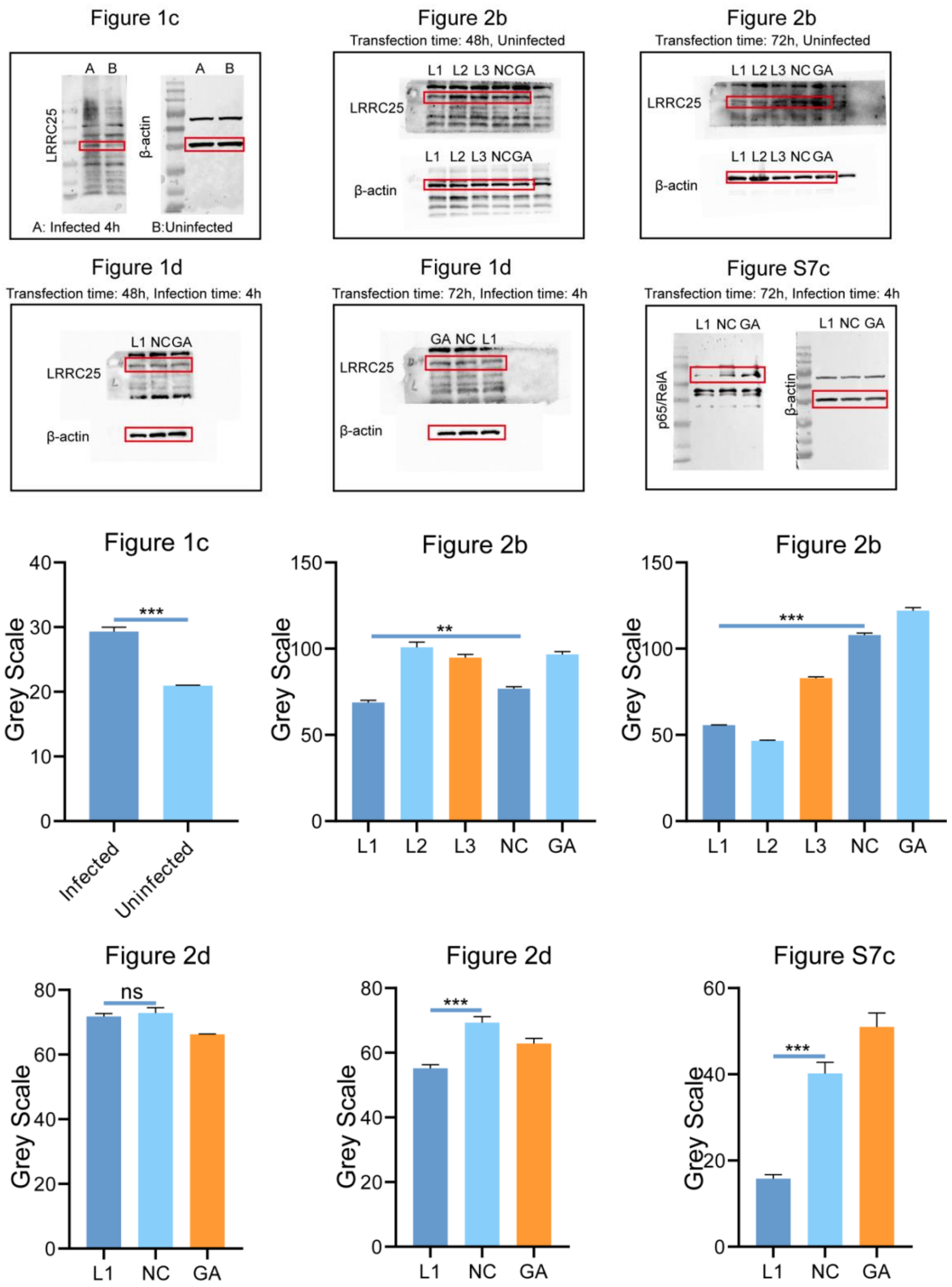

Figure S10. All the unprocessed WB original images and expression intensity in this study. Bar graphs show the mean ± SD form three independent experiments. Figure 1c: Student's t test. Figure 2b, 2d, S7c: One-way ANOVA. *$P < 0.05$, **$P < 0.01$, ***$P < 0.001$.

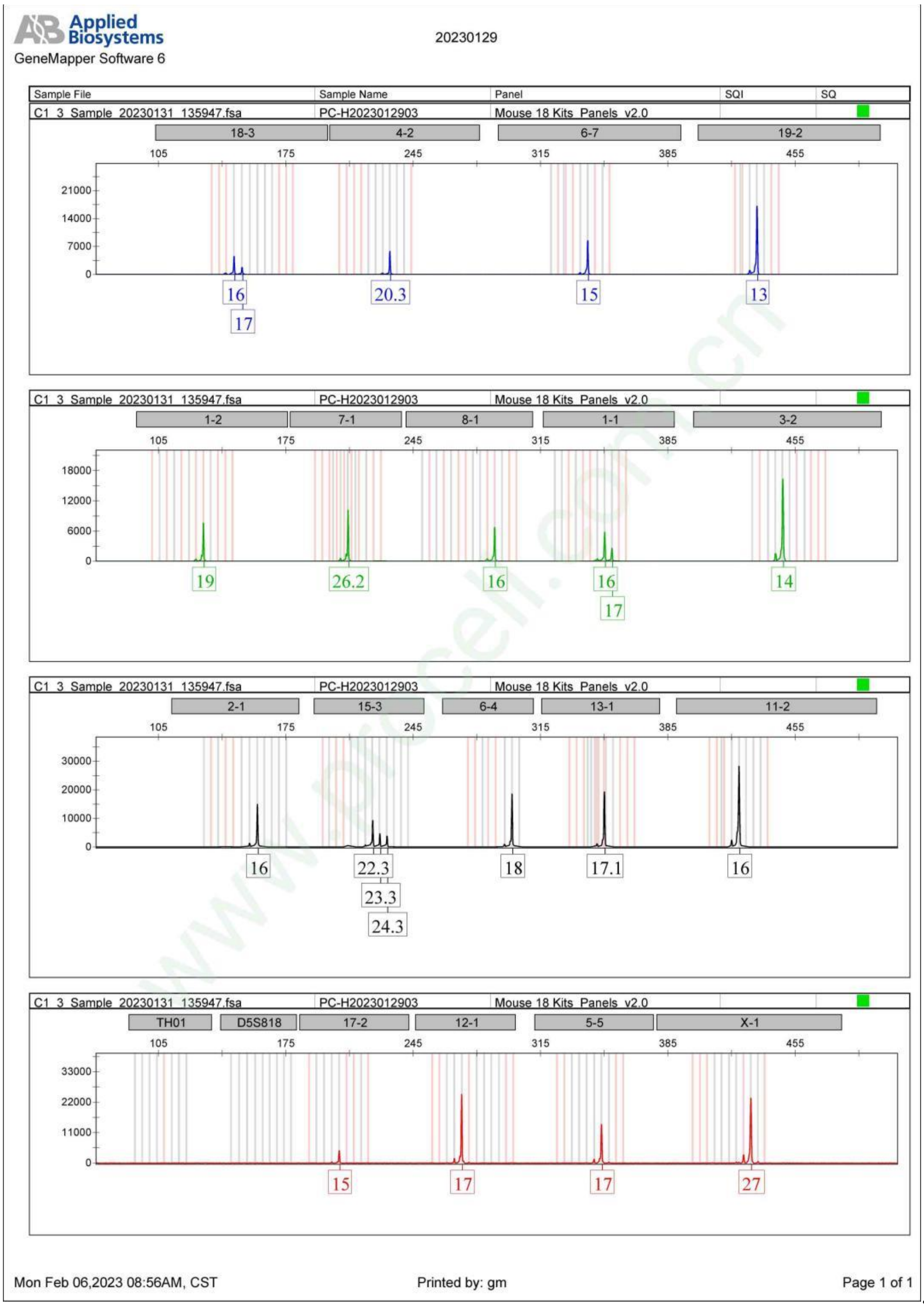

Figure S11. Short Tandem Repeat (STR) identification report of BV2 cells (Provided by Pricella).
